# A novel 268 kb deletion combined with a splicing variant in *IL7R* causes of severe combined immunodeficiency in a Chinese family: a case report

**DOI:** 10.1186/s12920-023-01765-8

**Published:** 2023-12-11

**Authors:** Lulu Yan, Yan He, Yuxin Zhang, Yingwen Liu, Limin Xu, Chunxiao Han, Yudan Zhao, Haibo Li

**Affiliations:** 1 The Central Laboratory of Birth Defects Prevention and Control, Ningbo Women and Children’s Hospital, Ningbo, Zhejiang 315000 China; 2 Department of Pediatrics, Ningbo Women and Children’s Hospital, Ningbo, Zhejiang 315000 China

**Keywords:** *IL7R*, Severe combined immunodeficiency, Whole exome sequencing, Chromosome microarray analysis, Splicing variant

## Abstract

**Background:**

Severe combined immunodeficiency (SCID) is a group of fatal primary immunodeficiencies characterized by the severe impairment of T-cell differentiation. *IL7R* deficiency is a rare form of SCID that usually presents in the first months of life with severe and opportunistic infections, failure to thrive, and a high risk of mortality unless treated. Although recent improvements in early diagnosis have been achieved through newborn screening, few *IL7R*-related SCID patients had been reported in the Chinese population.

**Case presentation:**

Here, we retrospectively analyzed a case of SCID in a 5-month-old girl with symptoms, including severe T-cell depletion, recurrent fever, oral ulcers, pneumonia, hepatosplenomegaly, bone marrow hemophagocytosis, and bacterial and viral infections. Whole-exome sequencing (WES), quantitative PCR (qPCR), and chromosome microarray analysis (CMA) were performed to identify the patient’s genetic etiology. We identified a 268 kb deletion and a splicing variant, c.221 + 1G > A, in the proband. These two variants of *IL7R* were inherited from the father and mother.

**Conclusions:**

To our knowledge, this is the first report of whole *IL7R* gene deletion in combination with a pathogenic splicing variant in a patient with SCID. This deletion also expands the pathogenic variation spectrum of SCID caused by *IL7R*. The incorporation of exome-based copy number variant analysis makes WES a powerful molecular diagnostic technique for the clinical diagnosis of pediatric patients.

## Background

Severe combined immunodeficiency (SCID) is a life-threatening condition leading to early infant death as a result of severe infection, due to impaired T lymphocyte differentiation [1, 2]. The prevalence of SCID is approximately 1 in 58,000 live births [3]. Early diagnosis before the onset of severe infections is key to the successful management of SCID, and prompt treatment in the first year of life results in the best outcomes [4, 5]. Hematopoietic stem cell transplantation is currently the standard treatment [6]. SCID is genetically heterogeneous, and defects in more than 30 genes have been identified to be causative of SCID, including defects in genes involved in antigen receptor gene rearrangement, T-cell receptor signaling, T-cell differentiation, thymic development, and thymic egress of T-cells [7, 8]. Biallelic variations in the *IL7R* gene abolish T cell development and function, resulting in SCID. *IL7R* deficiency causes T^–^B^+^NK^+^ SCID, which is responsible for 10% of the SCID cases [9–11]. Over 60 patients with SCID with *IL7R* deficiency have been reported worldwide since it was first described by Puel et al. in 1998 [2, 9, 10, 12–23].

The human *IL7R* gene is located at 5p13.2 and consists of two subunits, the IL7R alpha chain (IL7Rα) and common gamma chain (γc). It is expressed on lymphoid cells and plays an important role in the development, survival, homeostasis, and proliferation of T cells [24, 25]. Currently, in addition to the detection of conventional small insertions/deletions (indels) and single nucleotide variants (SNVs), exome-based copy number variant (CNV) analysis makes whole exome sequencing (WES) a powerful tool for the clinical diagnosis of genetic diseases [26, 27]. Herein, we retrospectively identified compound heterozygous variants in *IL7R*, with a maternal splicing variant, c.221 + 1G > A, and a paternal 268 kb deletion at 5p13.2 in a Chinese patient with SCID.

## Case presentation

**Fig. 1:**
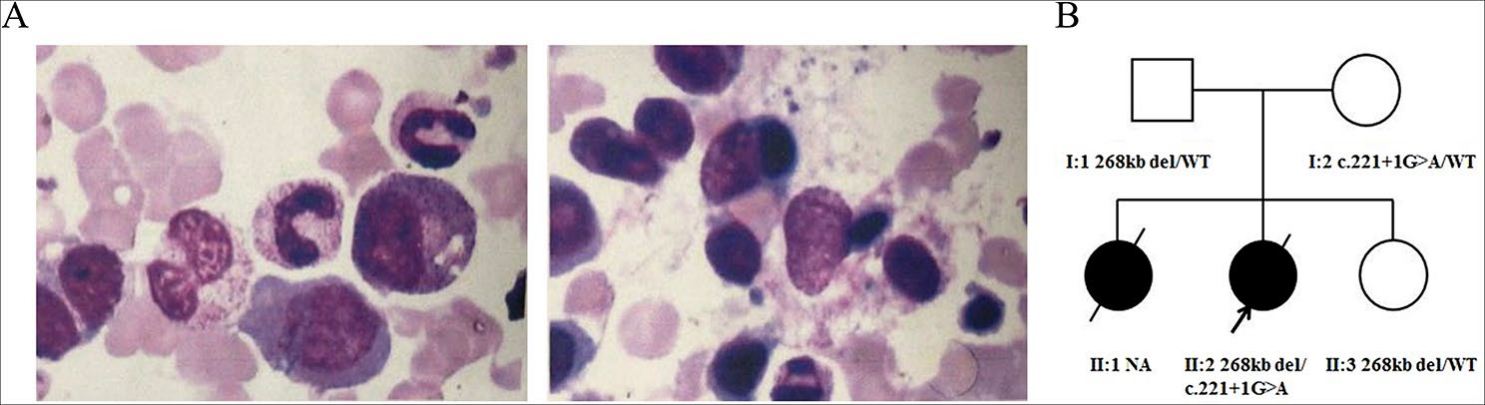
**A** Chinese family presents severe combined immunodeficiency (SCID). **A** Bone marrow aspirate smear with features of hemophagocytosis showing neutrophils engulfed by macrophages (Jenner Giemsa, ×400). **B** Pedigree of the family with SCID. Circles and squares indicate females and males respectively; filled symbols represent affected individuals with SCID; slashed symbols are deceased members; open symbols represent healthy individuals. Black arrow indicates the proband. The proband’s unaffected mother and father are carriers of the splicing variant and deletion respectively. WT, wild-type; ND, not determined

The proband (II2) was deceased in May 2011, but there were stored dried blood spots. The proband was a girl, the second daughter of non-consanguineous parents. She was a product of a full-term pregnancy via cesarean delivery, and her birth weight was 3.05 kg. She presented at age 3 months with yellow discoloration of the skin and sclera, tan urine, recurrent fever, oral ulcers, and pneumonia. Physical examination revealed that the patient’s spleen was 2 cm below the left costal margin in the midclavicular line with a soft and sharp margin, and the liver was 2.5 cm below the right costal margin in the mid-clavicular line. Laboratory examinations revealed a white blood cell count of 10.6 × 10^9^/L, with 88% neutrophils and 6% lymphocytes, a low hemoglobin level (8.6 g/dL), a high platelet count (815 × 10^9^/L), an elevated level of ferritin (732.5 ng/mL) and bilirubin (57.1 µmol/L), presence of Epstein-Barr virus and Rotavirus, and a positive result for direct anti-human globulin test. Additionally, bone marrow examination revealed active hemophagocytosis (Fig. 1A). She was treated with cefodizime, vancomycin, montmorillonite, and other symptomatic treatments; however, her fever did not subside. The patient died of severe bacterial sepsis two months later. Her older sister (I1) had clinical manifestations similar to those of the proband (recurrent febrile episodes, cough, severe anemia, and abdominal neoplasms), and died at seven months of age.

Dried blood spots were obtained from the proband, and blood samples were obtained from the unaffected parents and siblings after obtaining informed consent. Genomic DNA was extracted from dried blood spots and peripheral blood leukocytes for WES using SureSelect Human All Exon V6 kits (Santa Clara, CA, USA) and a HiSeq2500 sequencer (San Diego, CA, USA). The pathogenic variants were assessed using a protocol issued by the American College of Medical Genetics and Genomics (ACMG) [28]. Co-segregation analysis combined with bioinformatics analysis was used to validate the disease-causing variants.

**Fig. 2:**
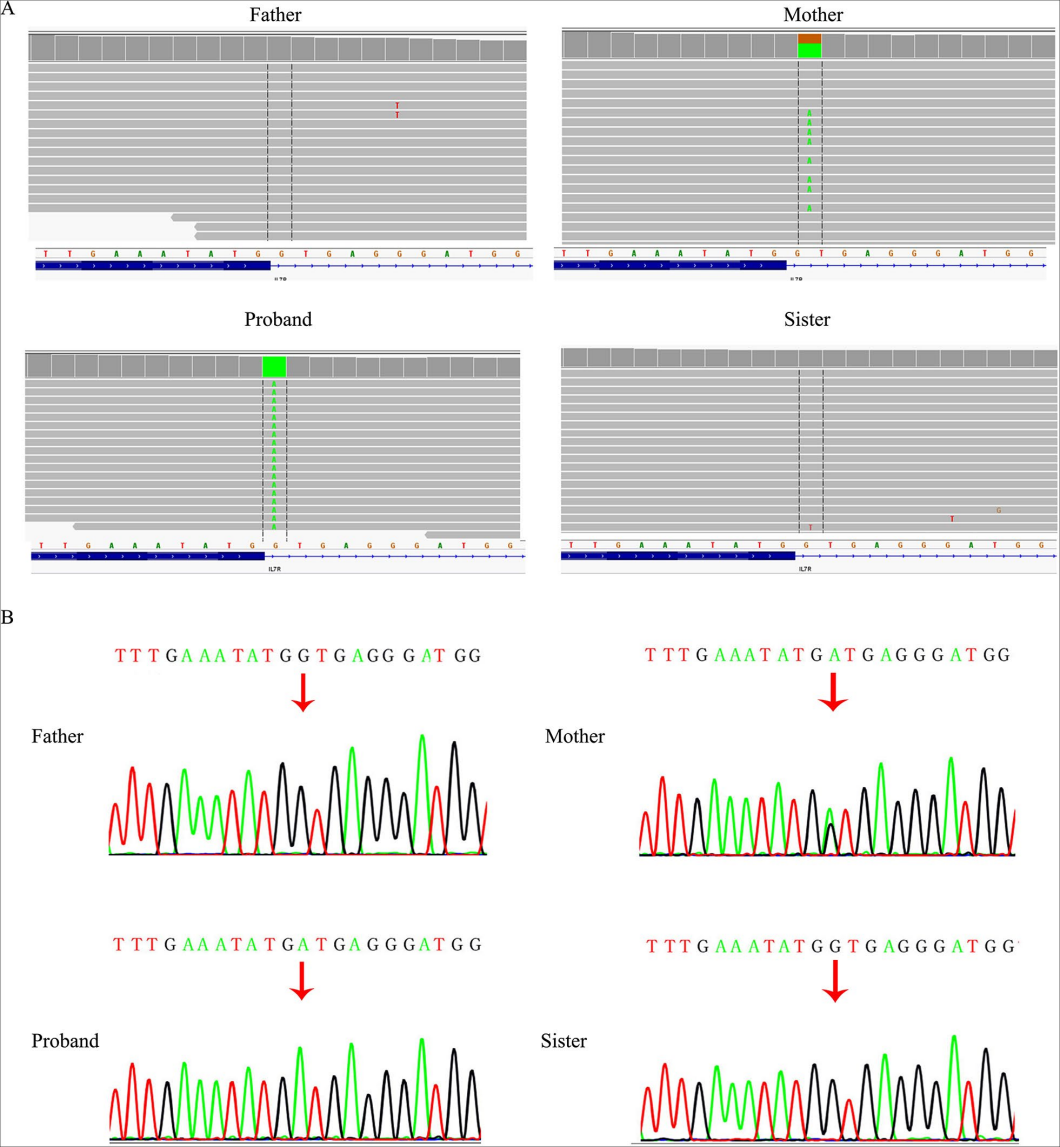
Identification of a splicing variant in the *IL7R* gene. **A** Whole-exome sequencing (WES) identified the homozygous variant c.221 + 1G > A in *IL7R* gene (NM_002185.5) in the proband viewed on Integrative Genomics Viewer. Her mother is a carrier of the variant. **B** Sanger sequencing confirmed a homozygous splicing variant c.221 + 1G > A in the proband. Her mother is a heterozygous variant carrier. Both her father and sister are homozygous for the wild-type allele. The variation site is marked by a red arrow

**Fig. 3:**
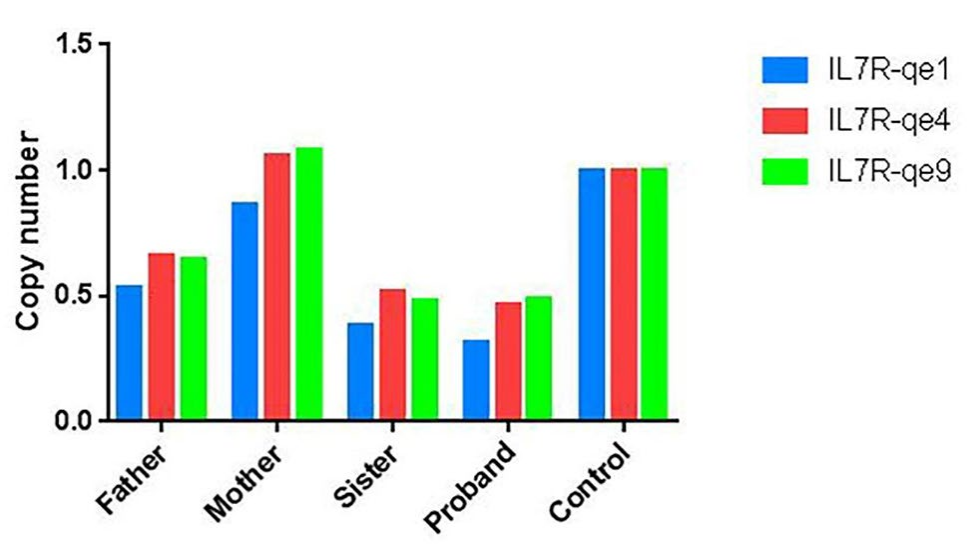
qPCR (copy number calculated) illustrating the *IL7R* gene deletion in the family. IL7R-qe1, IL7R-qe4, and IL7R-qe9 are three distinct regions of *IL7R* gene. Bar graph shows the calculated copy numbers of DNA from the proband, her father, mother, sister, and normal control by qPCR. The copy number decreased by half in the proband, her father, and sister as compared to the normal control, suggesting a heterozygous deletion of *IL7R* gene

**Fig. 4:**
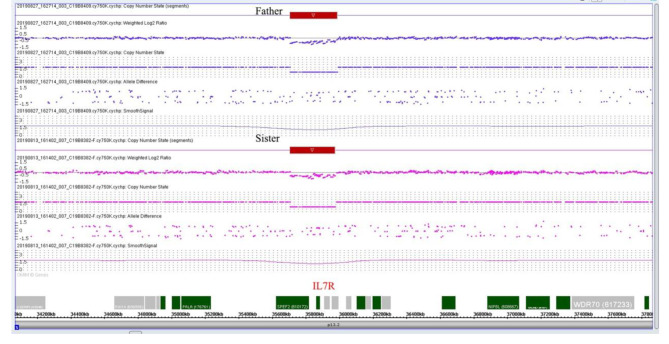
Chromosome microarray analysis results of the sister and father revealed a 286 kb heterozygous deletion (chr5:35,701,848 − 35,969,375) involving the *IL7R* gene located at 5p13.2. The deletion is identified by a downshift of the probes in the deleted region

WES revealed that the proband (II:2) had a novel homozygous variant in *IL7R* (NM_002185.5), c.221 + 1G > A, which was related to SCID and was previously reported in a Chinese infant [23]. We excluded pathogenic variants of other SCID-associated genes in the European Society for Immunodeficiencies guidelines. According to the ACGM classification guidelines for sequence variants, the variant c.221 + 1G > A was classified as pathogenic (PVS1, PM2_Supporting, PM3_Supporting, PP4). Her mother carried the heterozygous splicing variant, while her father (I1) and sister (II3) carried the wild-type alleles (Fig. 1B). This finding was confirmed by Sanger sequencing (Fig. 2A, B). The homozygosity of the c.221 + 1G > A variant in the proband could not be explained unless the other allele was lost. Thus, we analyzed potential CNVs of the *IL7R* gene using WES. Notably, the results suggested that the proband, her father, and sister may have a heterozygous deletion of the *IL7R* gene (NM_002185.5: chr5:35700839-35968226del). To further confirm the deletion of the *IL7R* gene, we performed qPCR. qPCR was performed to amplify *IL7R* exon 1, exon 4 and 8-noncoding region boundaries with three primers sets (Table 1). The *TERT* gene was chosen as the endogenous control in this study. All reactions using SYBR Green Dye were run using the following cycle: 30s at 98 °C, followed by 30 cycles of 10s at 98 °C, 20 s at 56 °C, 30s at 68 °C, and a final incubation at 68 °C for 7 min. All reactions were performed using the ABI 7500 real-time PCR system (Applied Biosystems, USA). All the experiments were replicated three times to ensure the accuracy of the experiment. After qPCR was performed, the copy number data were collected and analyzed using the 2^−ΔΔCT^ method. The qPCR results showed that the patient, her father, and her sister had approximately half of copy numbers for exons 1, 4, and 8-noncoding region boundaries of *IL7R* compared to those in the control, suggesting that one of the *IL7R* alleles was entirely deleted. To examine the *IL7R* heterozygous deletion, we performed chromosome microarray analysis (CMA) on the father and sister (the proband did not have enough DNA samples for CMA) (Fig. 3). The CMA results revealed a 268 kb heterozygous deletion (chr5:35,701,848 − 35,969,375) on 5p13.2 in samples of the father and sister (Fig. 4). None of the samples exhibited other pathological CNVs, areas of loss of heterozygosity or maternal uniparental disomy. Deletions can be classified as uncertain significance by the ACMG and the Clinical Genome Resource (ClinGen) [29]. The heterozygous deletion in the sister was inherited from her father, suggesting that the proband could have inherited the same 268 kb deletion from her father. Therefore, a homozygous splicing mutation is a heterozygous maternal mutation that is unmasked by a heterozygous paternal deletion.

**Table 1:** Primers used in qPCR

Region	Location	Name	Sequence ($5^{\prime}\rightarrow3^{\prime}$)
Exon 1	chr5:35857032–35,857,137	*IL7R*-qe1F	CATACACACTGGCTCACACA
		*IL7R*-qe1R	CAGAAACGACTTGAAGTAAAGA
Exon 4	chr5:35871182–35,871,288	*IL7R*-qe4F	TGAGTGTCGTCTATCGGGA
		*IL7R*-qe4R	GCGGTAAGCTACATCGTGC
Exon8-noncoding region boundaries	chr5:35877593–35,877,687	*IL7R*-qe9F	CTGCTACCACCCAACTGC
		*IL7R*-qe9R	GGTCATGCCTCCTCTCACT

## Discussion and conclusion

Currently, the combination of pathogenic variant screening and CNV calling has been increasingly used in WES, which largely improves the detection efficiency for the diagnosis of inherited diseases [30, 31]. Recent technological advances have enabled CNV calling from WES data using accurate and highly sensitive bioinformatics tools [32, 33]. Here, we identified a novel 268 kb deletion in the *IL7R* gene combined with a splicing variant, (c.221 + 1G > A), as the cause of the SCID phenotype in a female patient. The deletion was paternally inherited and the c.221 + 1G > A variant was maternally inherited.

**Fig. 5:**
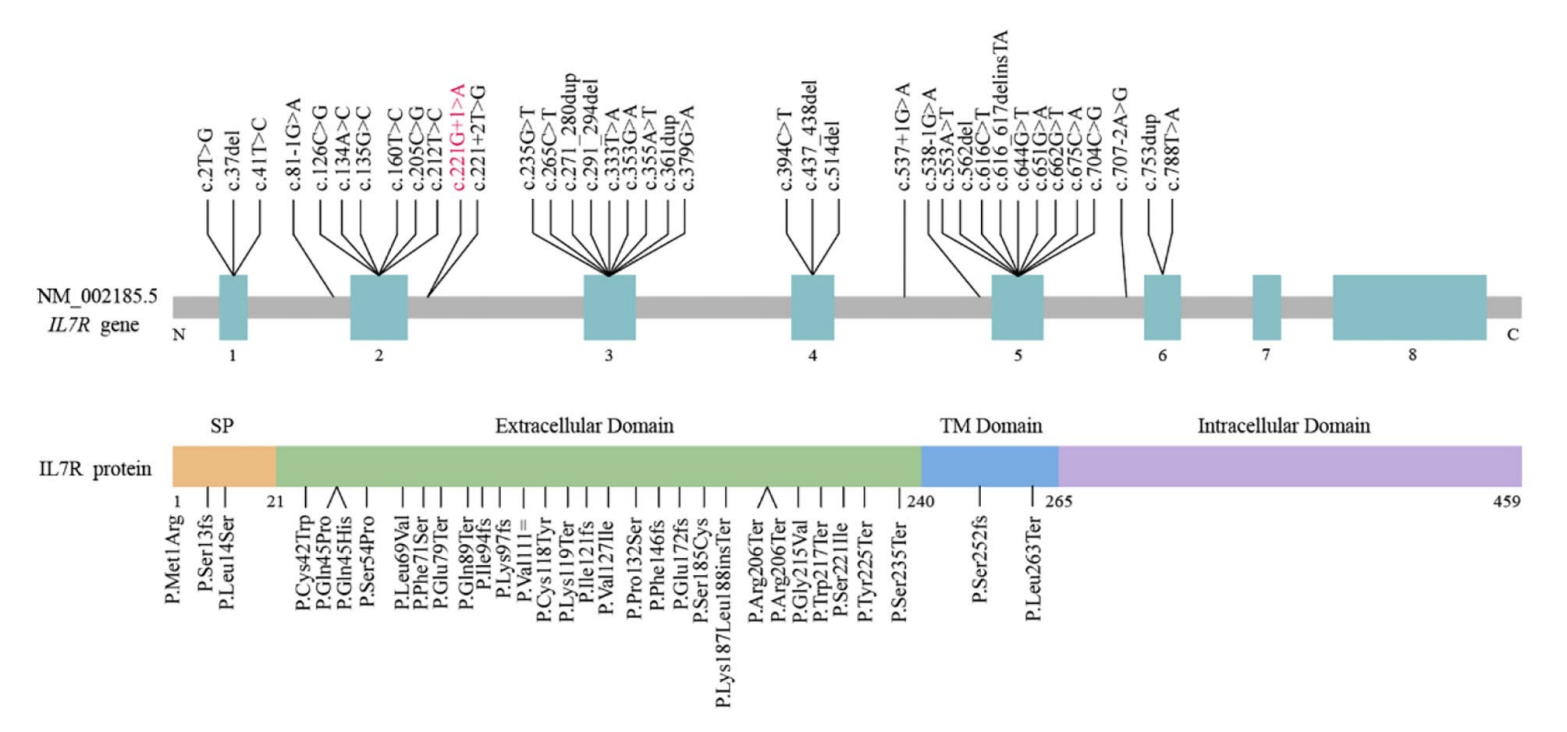
Distribution of IL7R domains and all likely pathogenic and pathogenic variants associated with SCID in ClinVar database. The reported variants are indicated in red (https://www.ncbi.nlm.nih.gov/clinvar/, the last access time was October 4, 2023)

The likely pathogenic and pathogenic variants of *IL7R* associated with SCID in the ClinVar database included 14 missense, 10 nonsense, seven frameshift, six splicing variants, and one synonymous variant (Fig. 5). The c. 221 + 1G > A splicing variant detected in our patient also has been included in the ClinVar database. IL7R contains three major domains: a transmembrane, an intracellular, and an extracellular domain. The extracellular domain is important for binding to IL-7 and belongs to one of the variable loop regions in the protein structure that is assumed to control the binding specificity of IL7R [34, 35]. Mutated IL7R may impair IL-7 signal transduction in T cell and cause T-cell deficiency.

The *IL7R* gene contains eight exons. Previously identified single- or multi-exon deletions in *IL7R* are common in T-B + NK + SCID and detectable by WES analysis. We identified 8 cases of the exon deletions of *IL7R* detected SCID through a literature review, approximately 100% of T-B + NK + patients with SCID had compound heterozygous *IL7R* deletions (Exon3del, Exon2-4del), and exon 3 is a deletion hotspot in *IL7R* [17, 21]. Notably, in our study, we reported a heterozygous 268 kb deletion that contained the entire *IL7R* gene in a patient with SCID, emphasizing the importance of identifying CNVs of *IL7R* in such cases. Furthermore, the 268 kb deletion in the patient also involved three genes adjacent to *IL7R*, *SPEF2, CAPSL* and *UGT3A1*, the heterozygous deletion of which did not cause a dominant genetic disorder.

Patients with SCID and impaired *IL7R* function may have immune dysregulation, severe infections, chronic inflammatory diseases, as well as cancer [36]. The main clinical manifestations in the older sister in this study were severe anemia and abdominal neoplasms. However, the proband presented with the typical clinical symptoms of SCID, including recurrent infections, fever, pneumonia, oral ulcers, and bacterial and viral infections. Additionally, the proband presented with atypical symptoms, including hyperferritinemia, hepatosplenomegaly, and bone marrow hemophagocytosis, which overlapped with the symptoms of hemophagocytic lymphohistiocytosis (HLH). HLH-like symptoms have not been previously described in SCID cases involving the *IL7R*. The atypical symptoms presented by our patient highlight the diagnostic challenges in the field of SCID. Typically, patients with complete loss of IL-7R present early onset SCID with profound T-cell lymphopenia and normal B and NK cell levels [10]. Unfortunately, we were unable to assess the lymphocyte levels of the patient without an immunological test because she died of bacterial sepsis. To the best of our knowledge, this is the first report of deletion of the entire *IL7R* allele in a Chinese family with SCID. We confirmed that the pathogenic variant c.221 + 1G > A in one *IL7R* allele is revealed by a 268 kb 5p13.2 deletion of the other allele. The simultaneous detection of SNVs/indels and CNVs in *IL7R* demonstrates the advantage of WES for identifying pathogenic variants and the importance of parental verification.

In conclusion, our patient received a definitive genetic diagnosis of SCID after her death, which was due to a compound heterozygous for the *IL7R* gene with a novel 268 kb deletion and a splice variant. This deletion also expands the pathogenic variation spectrum of SCID caused by *IL7R*. Further functional validation is necessary to clarify the pathogenesis of the *IL7R* gene in SCID.

## Data Availability

The detected variants have been submitted to the leiden open variation database (LOVD), at the following link: https://databases.lovd.nl/shared/individuals/00437939.

## Abbreviations

SCID: Severe combined immunodeficiency
WES: Whole exome sequencing
qPCR: Quantitative PCR
CMA: Chromosome microarray analysis
CNVs: Copy number variants
Indels: Insertions/deletions
SNVs: Single nucleotide variants
ACMG: American College of Medical Genetics and Genomics
HLH: Hemophagocytic lymphohistiocytosis
